# Chronic Diseases and Self-Reported Health Status Among American Indian/Alaska Native Older Adults

**DOI:** 10.1093/geroni/igab046.2382

**Published:** 2021-12-17

**Authors:** Ramona Danielson, Collette Adamsen, Agnieszka Mason

**Affiliations:** 1 North Dakota State University, Fargo, North Dakota, United States; 2 University of North Dakota, Grand Forks, North Dakota, United States

## Abstract

Background: In the 1800s and 1900s, U.S. federal “Indian” policy (e.g., boarding schools, relocation) created historical trauma with impacts that reverberate today, such as the significant health challenges experienced among American Indian/Alaska Native (AI/AN) populations. Our study seeks to better understand the burden of chronic disease, and also resilience, among AI/AN older adults. Methods: Data came from Cycle VII (2018-2020) of the National Resource Center on Native American Aging’s “Identifying Our Needs: A Survey of Elders” survey of AI/AN adults ages 55+ from primarily rural tribal survey sites (N=20,642). Analysis explored self-assessed health status (very good/excellent, good, fair/poor) and looked for significant differences in prevalence of chronic conditions a doctor ever told them they had (e.g., high blood pressure, diabetes, depression, arthritis, asthma). Results: Self-reported health among AI/AN adults age 55+ was: 26% very good/excellent, 39% good, and 35% fair/poor. 87% of respondents had 1+ chronic illness; 37% had 3+. Among those reporting very good/excellent health, 75% had 1+ chronic illness and 19% had 3+. High blood pressure was the most common chronic disease, at 56% (44% for very good/excellent compared to 67% for fair/poor), followed by diabetes, at 36% (24% for very good/excellent compared to 46% for fair/poor). Conclusions: All of the chronic conditions examined showed significantly higher prevalence among AI/AN adults 55+ with fair/poor health. Notably, 1 in 5 respondents with 3 or more chronic conditions indicated very good/excellent health, reinforcing that successful aging can still be experienced by those with chronic health conditions.

